# Supplementary material for: The Colombian scientific elite—Science mapping and a comparison with Nobel Prize laureates using a composite citation indicator
Source: PLoS One. 2022 May 26;17(5):e0269116. doi: 10.1371/journal.pone.0269116 (PMC9135245; doi:10.1371/journal.pone.0269116)
Supplement: Attachment — Submitted filename: letter of response.docx [file pone.0269116.s003.docx]

**Round 2 §**

Dear editor, reviewers—

The authors appreciate immensely the comments and suggestions for improving the article. Please, find below the response to each comment according to each reviewer. The response has three columns, one for each reviewer’s set of comments. The table was divided into paper sections (introduction – lit review; methods; results, and so forth). Additions to the article were highlighted in yellow. The new or modified sections added to the manuscript were edited —once again—by a professional proofreader.

The permanent link to the available data was also activated: <https://doi.org/10.34848/GJO6SY>

- Datasets:
  - List of the AAEP awarded 1990-2020 (xlsx format): lead author awarded-AAEP; author sex; year; award category; document title; abstract (Spanish); affiliation at the time the award was received; institution’s country and city; highest/last degree; and the university that granted the academic degree and country.
  - Processed matrices for the following science mapping networks (csv format):
    - Coauthorship matrices at the institutional and author levels by AAEP category: six matrices.
    - Bibliographic coupling matrices by AAEP category: three matrices.

Sincerely,

The authors.

Reviewer 1

Abstract

1) The abstract needs a full revision. It is not written as an abstract that should address what was done and the main conclusions drawn from the results in short.

Authors response

- *We edited the abstract as follows:*

A well-established agenda on the research output, impact, and structure of global scientific elites such as Nobel Prize laureates has generated interest in the scientific elites from developing countries. However, this topic has not been investigated in detail. This study, first, deploys science mapping techniques to provide a comprehensive analysis of the output, impact, and structure of the Colombian scientific elite, i.e., researchers awarded with the *Alejandro Ángel Escobar Foundation National Prize* 1990-2020*,* known locally as the *Colombian Nobel*. Second, this study also conducted a productivity and impact comparison between the Colombian scientific elite and Nobel Prize laureates in science and economics by means of a stratified random sample 1990-2020 via the composite citation indicator proposed by Ioannidis et al. Findings showed that the Colombian scientific elite has a broader agenda than indexing titles in internationally renowned bibliographic databases. The Colombian scientific elite also showed positive growth, which is an inverse trend compared with the sample of Nobel laureate productivity. There were no noticeable changes in productivity/impact before and after receiving the *Alejandro Ángel Escobar Foundation National Prize*. Institutional collaboration within the Colombian scientific elite displayed the highest betweenness (*brokerage*) role of world/local top-tier universities. However, only two Colombian scientific elite members published an article with two Nobel Prize laureates. Most of the research profiles reflected the national output priorities, but were found to diverge from the national focus in respect of strategic research capacities. The interleaving of the Colombian scientific elite and Nobel Prize laureates — particularly between the 3^rd^ and 2^nd^ quartiles — enabled a more nuanced analysis of the local impact in the global scientific landscape. Our findings also contrast with previous findings on the lower research impact of authors from Latin America, despite their involvement as contributors to reputable journals, and also shed light on the research performance-impact standards and agenda between the global North and South and provide an in-context assessment of outstanding local research.

Introduction

2) Rewrite the sentence on page 9 line 63: “Colombia does, however, have its own Nobel Prize:” it is not a “Nobel prize” since the prize is affiliated to a distinct institution and may have other forms of choosing their winners. Something like a Nobel equivalent should be more adequate.

Authors response

- *We edited the sentence as follows:*

Colombia does, however, have its own *Nobel Prize* equivalent: the *Alejandro Ángel Escobar Foundation National Prize* (AAEP) [46,47].

Results

3) Page 65 Table 3 first line column H index is written 1,03. Is it the Hm index ou maybe 1030? Please correct.

Authors response

- *We corrected H index in Table 3. It is the journal H index, not the author’s:*

- *We also corrected Table 4, dot (.) for decimals, and comma (,) for thousands.*

**Round 1 §**

Dear editor, reviewers—

The authors appreciate immensely the comments and suggestions for improving the article. Please, find below the response to each comment according to each reviewer. The response has three columns, one for each reviewer’s set of comments. The table was divided into paper sections (introduction – lit review; methods; results, and so forth). Additions to the article were highlighted in yellow. The new or modified sections added to the manuscript were edited —once again—by a professional proofreader.

Sincerely,

The authors.

| **Reviewer #1** | **Reviewer #2** | **Reviewer #3** |
| --- | --- | --- |
| Introduction – Lit. review | | |
| N/A | An important objective of the present study, focusing on the Scientific Elite of Colombia, is to understand the performance and structure of research in developing countries and the existing levels of cooperation compared to the Global Scientific Elite. The study is quite original in presenting an analysis methodology that allows the comparison of the scope and reach of scientific production and the respective impacts of elite groups in a country in relation to Nobel laureates using the same evaluation structure. | 1. On page 3, l.82, the authors mention that the limitations of the study and future agenda would be presented in the end. Not that clear to me. I did not identify details on limitations.  2. On page 3, l.78-80, the authors write that “The methods and techniques implemented are coauthorship 79 networks, both at the institutional and author levels; bibliographic coupling; and a comparative sample of 82 researchers using the composite indicator proposed by Ioannidis et al. [52]”. Then, they describe a sample of 87 awardees. Please clarify.  5. For readers not familiar with scientific indicators for Colombia, it’s difficult to figure out the whole picture offered by this study. Data on GDP in research and development, number of active scientists, and others are missing. |
| Authors response | | |
| - *We appreciate the rev. #2 acknowledgments immensely.* - *We added a section in the discussion to point out more clearly the limitations of the study:*   Our study has several limitations. First, the geopolitical scope of the study is limited to Colombia. Second, we did not consider macro-economic variables related to science and technology activities, such as public/private R&D investment; research grants and scholarships; mentoring; or the correlation between an increased scientific workforce and its scientific impact on the CSE. Such variables could shed light on the potential role of confounding variables. Third, the bibliographic data sourced from Scopus is not a complete picture of the scholarly communication production in Colombia nor in Latin America and the Caribbean.   - *We clarify the composition of the sample of researchers, both AAEP and Nivel Prize laureates. Also, we draw a brief description of each method used:*   The methods and techniques implemented are coauthorship networks, both at the institutional and author levels; bibliographic coupling; and a comparative sample of 82 researchers (i.e., 41 AAEPs and 41 Nobel Prize laureates) using the composite citation indicator proposed by Ioannidis et al. [55]. Coauthorship networks map scientific collaboration, enabling researchers to identify the extent to which social connections contribute to the co-creation of knowledge. Bibliographic coupling discloses the clustering of research fronts, while the proposed composite citation indicator addresses total impact, normalized coauthorship, and author order based on six indicators at the author level. Section 4 presents the results to be discussed in section 5. Finally, section 6 presents the conclusions, limitations, and future agenda.   - *We added several descriptive indicators for the Colombian context in science, technology, and innovation and a brief comparison with high-income countries:*   This study focuses on Colombia, which ranks among the top five countries in Latin America in total document output and the top-fifty in total citations worldwide 1996-2019 [41]. Colombia’s national investment in science, technology and innovation activities is a mere 0.8% of the GDP (gross domestic product) (2015- 2020) [42]. Similarly, the country’s investment in R&D (research and development) has yet to surpass 0.4% of the GDP (2015-2020), which is below the Latin American average of 0.7% (2013) [42,43]. The Latin American region is also below the world average of 2.2% in R&D investment (2018) [44]. Regarding the scientific workforce, Colombia has 58 researchers per million inhabitants (male: 65%; female: 35%), compared with 260 in Mexico (male: 67%; female: 33%) or 1,206 in Argentina (male: 47%; female: 53%) [43]. At the other end of the spectrum, i.e., in the high-income countries, Denmark has 7,310 researchers per million inhabitants (male: 65%; female: 35%) and Finland 7,009 (male: 68%; female: 32%) [43]. This disparity is all the more striking when viewed in the context of Noble Prize laureates from Colombia, the subject of this study. | | |
| Methods | | |
| 1) For instance, they use a confusing nomenclature in Table 3, page 17 out of 44. There is no direct correlation between column names and the mentioned variables in the methodology section a few lines and pages above. It seems that they present confusing data with wrong words. For example, there are no Authors per article less than one! This name is mistaken (the English use too since it is Authors per article and not Authors per articles).  3) It is unclear where to classify all the presented parameters as Macro, meso, or micro. B_coup, hm, r_eff, authors per article? Which category to organize them? And why are those variables defined if not all of them are in a single table for comparison?  5) There are issues with the wrong choice of areas. For instance, Natural Sciences are such a massive cluster and have distinct areas that are too difficult to compare. Medical sciences with Physics cannot be a good choice of comparison. It is like comparing two countries, one with a population ten times higher than the other. The choice of the Award academy of area group classification is no excuse for carrying the analysis of those areas together. |  |  |
| Authors response | | |
| - *We agree with rev. # 1 regarding the descriptive bibliometric indicators presented in tables 2 and 3. We opted for two modifications. First, we merged both tables in one—enabling readers to look at a single glimpse of both sets of authors (Colombian and global, scientific elites). Second, the ‘authors per article’ could be a confusing indicator that adds rather marginal information. For instance, if the indicator computes an x value below zero (x<0) it seems that less than 1 author published an article. However, the correct reading is that —on average— the number of documents published surpassed the number of authors (i.e., authors are being more productive; or that the social capital of the authors of our sample remains static over time with increasing production). Considering that our composite index approach is a more refined technique to study author productivity, we dropped the column ‘authors per article’ from tables 2 and 3 and all its mentions in the manuscript. Table 2 is as follows:* Table 2 Scopus author profiles and bibliometric descriptive of the CSE and GSE aggregated and by category 1996-2020. **Award category** \| **Profiles** \| **Documents** \| **Articles** \| **Annual growth%** \| **Authors** \| **Citations per article** \| **Most frequent journal**; ***\|Alejandro Aangel Escobar National Prize - CSE*** \| \| \| \| \| \| \|; PhySci \| 19 \| 1,206 \| 1,025 \| 2.25 \| 1,776 \| 55.1 \| *Physical Review A - Atomic Molecular and Optical Physics*; EnvSci \| 14 \| 485 \| 439 \| 6.67 \| 735 \| 26.4 \| *Biotropica*; SoSci \| 10 \| 38 \| 31 \| <1 \| 21 \| 4.2 \| *Revista de Estudios Sociales*; **Total** \| **41** \| **1,731** \| **1,195** \| **2.97** \| **2,532** \| **28.6** \|; ***Nobel Prize – GSE*** \| \| \| \| \| \| \|; Chemistry \| 13 \| 2,944 \| 2,6 \| -2.71 \| 3,66 \| 126.2 \| *Journal of the American Chemical Society*; Economics \| 6 \| 334 \| 226 \| -0.39 \| 247 \| 98.2 \| *American Economic Review*; Physics \| 12 \| 2,517 \| 2,166 \| -0.31 \| 2,094 \| 61.9 \| *Japanese Journal of Applied Physics*; Physiology or Medicine \| 10 \| 1,211 \| 897 \| -5.16 \| 2,558 \| 113.2 \| *Proceedings of the National Academy of Sciences of the United States of America*; **Total** \| **41** \| **7,006** \| **5,889** \| **-2.14** \| **8,559** \| **99.9** \| Source: [48,53,68,78,79]. Abbreviations: EnvSci: environmental sciences and sustainable development; SoSci: social sciences and humanities; PhySci: physics and natural sciences. *Regarding the observation of the science mapping (clustering) between different disciplines/fields, such a technique has been implemented to identify research fronts and interconnection between highly interdisciplinary fields such as sustainability (sustainable development goals) and others—regardless of the size of the disciplines/fields. We clarified and added supporting evidence as follows:* It is also an extremely serviceable tool in the analysis of highly interdisciplinary fields and has been used to analyze SDGs (Sustainable Development Goals) [1], and the complete set of *Nature* journal publications spanning 150 years, ranging from the arts and social sciences to earth and space and clinical medicine. It is, therefore, an appropriate method for analyzing such a highly interdisciplinary category as the PhySci category of the AAEP [2]. 1. Nakamura M, Pendlebury D, Schnell J, Szomszor M. Navigating the Structure of Research on Sustainable Development Goals. Philadelphia, US: Institute for Scientific Information; 2019. Available: https://bit.ly/3qxC9v3 2. BarabasiLab 2019. On the shoulders of giants. In: Nature [Internet]. 6 Nov 2019 [cited 18 Jan 2022]. doi:10.1038/D41586-019-03165-4 *Rev. #1 pointed out a lack of clarity on our behalf regarding the macro, meso, micro levels of network analysis. This level-segmentation concerns only the science mapping sections. It has no relationship with the composite indicator section. We based our level-segmented approach on Yan E, Ding Y. Scholarly network similarities: How bibliographic coupling networks, citation networks, cocitation networks, topical networks, coauthorship networks, and coword networks relate to each other. J Am Soc Inf Sci Technol. 2012;63: 1313–1326. doi:10.1002/asi.22680. In that view, we clarify this approach as follows:* We used a multi-level approach for all the science mapping techniques implemented (i.e., coauthorship and bibliographic coupling analyses). A multi-level approach means analyzing the different structural features of a network at the macro (i.e., density and average path length); meso (i.e., community detection); and micro (i.e., betweenness centrality) levels through the use of network indicators [84]. Regarding the multi-level network indicators used, density indicates the degree to which both authors and institutions are connected (i.e., the number of connections that *exist* compared to the number of connections that *could* exist). - *We agree with rev.# 1 on methodological flaws comparing sciences/disciplines with the same yardstick. For example, there is no point in comparing the production/impact structure between quantum physics and anthropology counting full documents or citations. As rev. #1 points out, different sciences/disciplines have experimented with different historical path dependence, maturity, traditions, consensus/dissension, ontologies, epistemologies, incentives, funding, and so forth. In that view, we proceeded as follows:* - *We re-classified each researcher of the total sample (both Nobel Prize laureates, and AAEP winner) according to the disciplinary area of each researcher based on their research output—and not according to the awards category; we maintained the categories column in table X for the sake of identification of each elite group:* - We re-classified CSE and GSE researchers according to their core discipline/subject categories since awardees in respect of both prizes have a wide range of (under)graduate backgrounds. Moreover, prize categories themselves cover a wide range of disciplines/research areas. For instance, Juan Camilo Cárdenas, who belongs to the CSE, graduated from industrial engineering and was awarded his prize in the AAEP-EnvSci category (2009). Another example is Daniel Kahneman, a psychologist who received the Nobel Prize in economics (2002). The re-classification was conducted as follows: - Each CSE and GSE publication belongs to a single journal. Journals and serial titles are classified using the All Science Journal Classification (ASJC), which is based on the aims and scopes of the title and its content [93]. - We cross-checked the printed ISSN of each journal in which the CSE and GSE researchers have published and matched its ASJC subject area. This cross-check procedure was conducted for each CSE and GSE researcher. - If a given journal belonged to more than one ASJC subject area, it was randomly assigned. - We then computed each researcher’s publication frequency according to the ASJC subject areas. - Each researcher was then assigned to one ASJC subject according to its own most frequent ASJC subject based on its publishing record. For example, Cárdenas, an engineer who was awarded a prize in the EnvSci category, was assigned to the economics, econometrics and finance ASJC subject since most of his articles were published in journals under that classification. - *We added a new section presenting the methodological considerations for comparing different disciplines; and updated new research where the composite indicator has been used comparing individual researchers across research fields.* Since our sample is composed of researchers from multiple disciplines, we had to keep in mind the disciplinary differences so as not to commit methodological flaws, such as comparing the output and citations traditions and research dynamics between two researchers from physics and anthropology. For example, almost ¾ of research references in the humanities refer to books, whereas 80% of those in the natural sciences refer to journal articles [1]. Accordingly, we replicated multiple citation indicators and their composite proposed by Ioannidis et al. [2] to compare individual researchers across different fields. In their seminal study, Ioannidis et al. [2] computed the composite citation indicator (henceforth: *Ci*) for 84,000+ scientists from 12 fields (physics, mathematics, computer science, chemistry, earth sciences, engineering, biology/biotechnology, infectious disease, medicine, brain research, health sciences, and social sciences). *Ci* has also been used to compare software engineering with the multiple sub-fields of ‘information & communication technologies;’ to assess the scholarship of media experts on COVID-19 by gender and country; and to estimate the publishing output of COVID-19 and infectious disease experts across 174 research sub-fields [3–5]. 1. Gingras Y. Bibliometrics and research evaluation - Uses and abuses. Cambridge, Massachusetts: The MIT Press; 2014. 2. Ioannidis JPA, Klavans R, Boyack KW. Multiple Citation Indicators and Their Composite across Scientific Disciplines. PLoS Biol. 2016;14: 1–17. doi:10.1371/journal.pbio.1002501 3. Ioannidis JPA, Salholz-Hillel M, Boyack KW, Baas J. The rapid, massive growth of COVID-19 authors in the scientific literature. R Soc Open Sci. 2021;8. doi:10.1098/rsos.210389 4. Ioannidis JP, Tezel A, Jagsi R. Overall and COVID-19-specific citation impact of highly visible COVID-19 media experts: Bibliometric analysis. BMJ Open. 2021;11. doi:10.1136/bmjopen-2021-052856 5. Petersen K, Ali NB. An analysis of top author citations in software engineering and a comparison with other fields. Scientometrics. 2021;126: 9147–9183. doi:10.1007/s11192-021-04144-1 | | |
| Results | | |
| 4) There are also problems with graph presentation that uses lines to connect points that are uncorrelated through time; a large number of years that difficult reading them in an axis; comparison between large numbers and smaller ones that become flat in a scale should be made in a logarithm scale. |  | 8. Figures and Tables should be self-contained, as we know. Please revise those which are not. Example: “Table 6 Composite index (C) for the CSE and GSE” (page.25, l.519). |
| Authors response | | |
| - *We adjusted the plots following rev.#1 suggestion (log-10 for the y-axis; and smooth the figure lines):*        - *The description of the composite indicator requested by rev. #3 is located in the methods section. Also, below the Table mentioned, we describe the meaning of each abbreviation discussed, as follows:*   **Source: the authors based on** [55,93,102]**. Note: NC: total citations; H: H index; Hm: Schreiber Hm index; NS: total citations for papers where the scientist is the single author; NSF: total citations for papers where the scientist is the single or first author; NSFL: total citations for papers where the scientist is the single, first, or last author; C: composite citation indicator.** | | |
| Discussion | | |
|  |  | 3. On page 29, l. 570-571 the authors state that “Compared to the GSE ─ members of which received the Nobel prize at their peak, followed by a brief halo effect ─ there is no discernible halo effect for the CSE [22,106].”. [L. 576-578] They write that “It is also important to mention the lack of sustained and well-funded research in Colombia [107] ─ a constraint that is likely to persist, resulting in intermittent productivity and impact [108].” I think this lack of sustained funding makes this analysis a little bit complicated – especially because this is not usually the case for the countries of most Nobel prize winners. Please explain why your comparison to the GSE would be reliable despite this fator.  4. As Argentina has a marked role as a leading Latin American country in terms of Nobel prizes (https://eserb.cancilleria.gob.ar/en/2021-year-tribute-nobel-prize-medicine-dr-c%C3%A9sar-milstein), I missed a broader context for the discussion of the results. Please justify why Argentina is not mentioned in your discussion.  7. R. K. Merton and H. Zuckerman’s contributions could be more evident in the rationale of the study and discussion of results. They are cited but do not have an active part in the discussion for this particular Latin America scenario. Does the concept of scientific elite put forward by Zuckerman apply to the Colombian scientific elite? |
| Authors response | | |
| - *Following rev. #2 suggestions describe the Colombian STI (science technology and innovation) context in the introduction, highlighting the lack of financial and human resources dedicated to STI activities.* - *We did not consider pointing the Nobel Prize laurates from Argentina and other LATAM countries since the article's main purpose is to compare the CSE (Colombia) with the GSE. We mentioned some other LATAM institutions and their Nobel Prize alumni or affiliated researchers as long as their institutions figured in the institutional coauthorship analysis. Unfortunately, Argentinian institutions did not figure among the coauthorship networks.*   *As rev.#3 noticed, the study should be more transparent with its limitations. Therefore, we extended a section in conclusion in that regard.*   - *Regarding the R&D national funding and its relationship with the consolidation of a rather global or local scientific elite, we added this section:*   It is also important to mention the lack of sustained and well-funded research in Colombia [1] — a constraint that is likely to persist, resulting in intermittent productivity and impact [2]. National investment in science and technology activities, the leadership of public institutions, and the building of a secure and protected research environment within which ideas can grow and flourish are essential factors that contribute to outstanding research outcomes. For instance, between 2000-2008 the GSE reported a successful record in funding Nobel Prize-winning research by the National Institutes of Health and the National Science Foundation in the US [3]. Even when a substantial fraction of such research is unfunded, public and private research institutions provide a protected and safe environment for brilliant researchers to explore and innovate ideas [3].  1. OCyT. Indicadores de ciencia y tecnología. Bogotá, Colombia; 2020. Available: www.ocyt.org.co  2. Fortin J-M, Currie DJ. Big Science vs. Little Science: How Scientific Impact Scales with Funding. PLoS One. 2013;8: 1–9. doi:10.1371/journal.pone.0065263  3. Tatsioni A, Vavva E, Ioannidis JPA. Sources of funding for Nobel Prize‐winning work: public or private? FASEB J. 2010;24: 1335–1339. doi:10.1096/fj.09-148239   - We added a more than important remark by rev. #3 on the lack of discussion with the seminal work of Zuckerman on the scientific elites:   A final note regarding Zuckerman's pioneering work on the stratification in US Science and the CSE [9,10]. Zuckerman was particularly concerned about the growing inequality in the distribution of scientific recognition. We found evidence of this in the higher betweenness of institutions with a higher reputation at both local and global level, a reputation, moreover, that is substantially enhanced by having an AAEP awardee affiliated with such institutions. Zuckerman also pointed that —despite such inequality— ‘no scientist is deprived of all the good things of the scientific life’, including those in middle/lower positions [9]. We found this to be reflected in the eclectic and inclusive purpose of the AAEP that goes beyond the ‘*you must have a PhD to conduct research*’ or the ‘you must publish —research articles in English in top-tier journals— or perish.’ Lastly, Zuckerman lauded science’s egalitarian ideology for the way in which it brings together various scientist strata, an interweaving feature and impact in science that emerged in our analysis of *Ci* results for both the CSE and the GSE. | | |
| Conclusion | | |
|  |  | 1. On page 3, l.82, the authors mention that the limitations of the study and future agenda would be presented in the end. Not that clear to me. I did not identify details on limitations.  6. There are no comments on possible counfounders in the whole analysis. Could you please comment on them? |
| Authors response | | |
| - *We added a section in the discussion to point out more clearly the limitations of the study:*   Our study has several limitations, starting with the geopolitical scope limited to Colombia. In addition, we did not consider macro-economic variables related to science and technology activities, such as public/private R&D investment; research grants and scholarships; mentoring; or the increasing of the scientific workforce and their relationship with the scientific impact the CSE. Such variables could shed light on the potential role of confounding variables. Also, the bibliographic data sourced from Scopus’ is not a complete picture of the scholarly communication production in Colombia nor Latin America and the Caribbean.   - *Regarding confounding variables, we add restrictions and further agenda point to our study, as follows:*   Our study has several limitations. First, the geopolitical scope of the study is limited to Colombia. Second, we did not consider macro-economic variables related to science and technology activities, such as public/private R&D investment; research grants and scholarships; mentoring; or the correlation between an increased scientific workforce and its scientific impact on the CSE. Such variables could shed light on the potential role of confounding variables. Third, the bibliographic data sourced from Scopus is not a complete picture of the scholarly communication production in Colombia nor in Latin America and the Caribbean.  Further research could source other scientific elites (e.g., *Royal Society Africa Prize*, *Highest Science and Technology Award*, China; *Prêmio Almirante Álavaro Alberto*, Brazil), thereby deepening the understanding of research impact and structure in the context of developing regions/countries. Sourcing bibliographic data from search engines/databases with more comprehensive coverage — such as Google Scholar or Dimensions — could generate further insights by including researchers with no publications in either Scopus or WoS. The inclusion of macroeconomic variables relating to science and technology activities together with the influence of these activities on the impact of the CSE and the national scientific workforce in general could add a socio-economic dimension, thereby yielding a more comprehensive outlook. Lastly, the inclusion of *altmetrics* could provide another perspective: on elites that lie beyond the academic community, such as public debates conducted via social media. | | |
| References – Others – Editor requests | | |
| 2) Other English errors/mistranslated concepts are all over the manuscript, such as coauthorship adjustment that would be a normalized coauthorship. An adjustment would be a fitting (a better word to be used), but it does not contain the correct meaning.  6) References have some issues to I identified that reference 26 is repeated in 99. The is a ? in reference 98 and some internet sites that are not accessed recently. Authors should verify if they’re still active at the time of the submission. |  |  |
| Authors response | | |
| - *We changed (co)authorship adjustment for normalized (co)authorship throughout the manuscript.* - *We merged 26-99 references into one:*   Wagner CS, Horlings E, Whetsell TA, Mattsson P, Nordqvist K. Do nobel laureates create prize-winning networks? An analysis of collaborative research in physiology or medicine. PLoS One. 2015;10: 1–13. doi:10.1371/journal.pone.0134164   - *We double-checked the URLs and transformed the URLs into a short format for better presentation:*   The Nobel Foundation. All Nobel Prizes in Physics. 2020. Available: <https://bit.ly/3fvzADu>  SCImago. SJR - International Science Ranking. 2020 [cited 12 Jan 2021]. Available: <https://bit.ly/3Ih0qMa>  Clarivate Analytics. Recipients - Highly Cited. 2020 [cited 12 Jan 2021]. Available: <https://bit.ly/3nzaQi0>  Fundación Alejandro Ángel Escobar. Home. [cited 6 Aug 2020]. Available: <https://bit.ly/33pLeNG>  Quacquarelli Symonds. QS World University Rankings 2019: Top Global Universities. 2020 [cited 9 Jun 2020]. Available: <https://bit.ly/3qDUXZu>  Scopus. Scopus - Document search. 2020 [cited 1 Jun 2020]. Available: <https://bit.ly/3KhKoDn>  Scopus. Scopus Roadmap: What’s coming up in 2020 & 2021? 2020 [cited 1 Feb 2021]. Available: <https://bit.ly/3270UVw>  Nakamura M, Pendlebury D, Schnell J, Szomszor M. Navigating the Structure of Research on Sustainable Development Goals. Philadelphia, US: Institute for Scientific Information; 2019. Available: <https://bit.ly/3qxC9v3>   - *Following editorial policies, we made our data available (according to PLoS ONE third-party data policy). We added the following section in* ***‘Materials and methods’****:*   Data availability  In the permanent link that follows are publicly available the following datasets:   - Permanent link: <https://doi.org/10.34848/GJO6SY> **[link will be accessible after acceptance]** - Datasets:   - List of the AAEP awarded 1990-2020 (xlsx format): lead author awarded-AAEP; author sex; year; award category; document title; abstract (Spanish); affiliation at the time the award was received; institution’s country and city; highest/last degree; and the university that granted the academic degree and country.   - Processed matrices for the following science mapping networks (csv format):     - Coauthorship matrices at the institutional and author levels by AAEP category: six matrices.     - Bibliographic coupling matrices by AAEP category: three matrices. | | |
